# Development of Robust MWCNT Hydrogel Electrochemical Biosensor for Pyocyanin Detection by Phosphotungstic Acid Modification

**DOI:** 10.3390/s25020557

**Published:** 2025-01-19

**Authors:** Ting Xue, Lei Gao, Xianying Dai, Shenhui Ma, Yuyu Bu, Yi Wan

**Affiliations:** 1 Key Laboratory of Wide Band-Gap Semiconductor Materials and Devices, School of Microelectronics, Xidian University, Xi’an 710071, China; 22111213663@stu.xidian.edu.cn (T.X.); mashenhui@xidian.edu.cn (S.M.); 2 Microbiology Institute of Shaanxi, No.76 Xiying Road, Xi’an 710043, China; gaolei_work@163.com

**Keywords:** pyocyanin, electrochemical sensor, composite hydrogel, grotthuss effect, robust adhesion

## Abstract

The trace detection of pyocyanin (PCN) is crucial for infection control, and electrochemical sensing technology holds strong potential for application in this field. A pivotal challenge in utilizing carbon materials within electrochemical sensors lies in constructing carbon-based films with robust adhesion. To address this issue, a novel composite hydrogel consisting of multi-walled carbon nanotubes/polyvinyl alcohol/phosphotungstic acid (MWCNTs/PVA/PTA) was proposed in this study, resulting in the preparation of a highly sensitive and stable PCN electrochemical sensor. The sensor is capable of achieving stable and continuous detection of PCN within the range of 5–100 μM across a variety of complex electrolyte environments. The limit of detection (LOD) is as low as 1.67 μM in PBS solution, 2.71 μM in LB broth, and 3.63 μM in artificial saliva. It was demonstrated that the introduction of PTA can complex with PVA through hydrogen bonding to form a stabilized hydrogel architecture, effectively addressing issues related to inadequate film adhesion and unstable sensing characteristics observed with MWCNTs/PVA alone. By adjusting the content of PTA within the hydrogel, an increase followed by a subsequent decrease in sensing current response was observed, elucidating how PTA regulates the active sites and conductive network of MWCNTs on the sensor surface. This study provides a new strategy for constructing stable carbon-based electrochemical sensors and offers feasible assistance towards advancing PCN electrochemical sensors for practical applications.

## 1. Introduction

*Pseudomonas aeruginosa* (PA) is a Gram-negative pathogen, which is prone to infection in cystic fibrosis (CF), diabetic foot, and immunocompromised patients, and PA is also found to be the second most common pathogen in burn wound secretions [1]. Pyocyanin (PCN) serves as a distinctive marker for PA secretion, and quantitative analysis of PCN can determine the presence of PA infection. However, conventional analytical techniques such as high-performance liquid chromatography (HPLC) and spectrophotometry require sophisticated detection instruments and laborious pretreatment procedures, which are not suitable for real-time and rapid detection purposes [2]. The electrochemical sensing technology reflects the concentration of the substance to be measured by monitoring variations in electrical parameters, such as current and voltage, during the redox process, so as to achieve rapid detection and make up for the shortcomings of traditional detection methods [3]. PCN, also known as 1-hydroxy-5-methylphenazine, can undergo a reversible redox reaction by losing two electrons and gaining two protons, which makes quantitative detection of PCN possible using electrochemical techniques [4].

The unique characteristics of nanomaterials, including their large specific surface area, tunable electrical properties, and facile modification by chemical ligands and biomolecules, make them highly suitable for biosensing applications. Nanomaterials commonly utilized in the field of sensing include metal nanomaterials, metal compound nanoparticles, and carbon-based nanomaterials. Metal and metal compound nanomaterials exhibit excellent electrical conductivity and high sensitivity. However, they are prone to oxidation, possess poor chemical stability, and often require treatment with corrosive solutions, among other challenges [5,6]. Carbon-based materials, such as carbon quantum dots, carbon nanotubes (CNTs), and graphene, have well-established preparation processes, excellent stability, and low ohmic resistance that contribute to the advancement of electrochemical sensing electrodes [7,8]. Among these materials, CNTs can efficiently increase the electrochemical effective surface area of the electrode, promote the movement of charge carriers between the electroactive substance and the electrode, and immobilize proteins in electrochemical reactions, making them the best modified materials for preparing working electrodes and widely used in the field of biosensing. Multi-walled carbon nanotubes (MWCNTs) are composed of multiple layers of graphite that provide a larger surface area and facilitate binding to biomolecules for further detection sensitivity [9,10]. However, the weak interaction force between CNT dispersion and conductive substrate poses a challenge in achieving stable immobilization of CNTs on a conductive substrate.

Hydrogels are three-dimensional (3D) network structures formed by cross-linking polymer chains. Their excellent biocompatibility renders hydrogels widely used in biosensing, drug delivery, wound healing, and other fields. When compounded with a carbon-based material, it encapsulates the functional material within the hydrogel matrix to prevent detachment from the substrate [11]. Simultaneously, the hydrogel utilizes a mechanical interlocking effect to adhere to rough conductive substrates. Nevertheless, this mechanism may fail when binding to smooth substrates. Additionally, during the cross-linking crystallization process, hydrogels tend to shrink, leading to an accumulation of internal stress at the edges, which results in stratification of adhesive layers and disruption of adhesion effectiveness. A more significant issue is that the functional layer peels off after hydrogel swelling in a solution environment, significantly limiting the feasibility of nanocomposite hydrogels for applications [12,13].

The adhesion of hydrogels can be enhanced through surface modification [14,15], bridging polymers [16], topological adhesion [17], and incorporation of nanomaterials [18]. The first three methods involve introducing an intermediate layer between the hydrogel and substrate to form a closely adhesive sandwich structure facilitated by the intermediate layer. It is important to note that these methods do not directly improve the inherent adhesion ability of the hydrogel itself. Furthermore, incorporating such intermediate layer substances may compromise biocompatibility and impose limitations on usage conditions (e.g., pH and temperature). However, the mechanism of action of nanoparticles is to complex with polymers through their surface functional groups, change the polymer structure and fragmentation dynamics, and achieve tight adhesion with the substrate. Moreover, nanoparticle composite hydrogels can enhance electrical conductivity and antimicrobial properties, expanding hydrogel applications in sensing [19,20,21,22]. Polymetallic oxides (POMs) are clusters of metal oxides at the nanoscale. Phosphotungstic acid (PTA) exhibits a typical Keggin structure wherein oxygen atoms possess multiple bonding modes capable of forming hydrogen bonds with polyvinyl alcohol (PVA), an ideal material for hydrogels. This interaction reduces PVA crystallinity while attenuating internal stress during the film curing process and promoting the formation of a robust adhesive structure with the substrate [23].

Therefore, MWCNTs/PVA/PTA nanocomposite hydrogels were developed via self-assembly in this study to address the challenge of insufficient adhesion between hydrogels and smooth substrates, which hinders continuous detection of target substances. And electrochemical sensors based on MWCNTs/PVA/PTA were synthesized. The content of the composite PTA was controlled to investigate its role in the conductive network mechanism and its impact on sensing performance. The results indicate that the proportion of PTA in the hydrogel is a crucial factor in regulating the surface morphology and sensing performance of the electrochemical sensor. Optimal incorporation of PTA can diminish the crystallinity of PVA, enhancing both adhesion capacity and accessible active sites while improving sensing sensitivity. The MWCNTs/PVA/PTA4 sensor is capable of linearly detecting PCN within a range of 5–100 μM in various electrolyte environments, with a limit of detection (LOD) of 1.67 μM. And the sensor has certain anti-interference, stability, and reproducibility.

## 2. Experimental Section

### 2.1. Materials and Instruments

The details of materials and instruments used in this work are provided in the Supporting Information.

### 2.2. Preparation of the MWCNTs/PVA/PTA Electrochemical Sensor

A homogeneous dispersion of MWCNTs with a mass concentration of 0.01 g/mL was prepared by dispersing 0.1 g of carboxyl polar group functionalized MWCNTs into 10 mL of SDS aqueous solution (0.01 g/mL), stirring at 1000 rpm for 30 min, and then subjecting the solution to ultrasonic treatment for 1 h. Subsequently, 0.5 g of PVA was added to the aforementioned dispersion and stirred in a water bath at 95 °C for 1 h to ensure complete dissolution of PVA. The water bath environment was cooled to 50 °C, followed by the addition of PTA, and the mixture was continuously stirred at the same speed for 30 min. Different ratios of MWCNTs/PVA/PTA composite hydrogels were successfully prepared based on varying amounts of added PTA, and these hydrogels were sealed and stored under ambient conditions. The different hydrogel systems were expressed according to the mass of PTA added during the preparation process. MWCNTs/PVA/PTA2 denotes the addition of 0.1 g of PTA to the MWCNTs/PVA hydrogel; MWCNTs/PVA/PTA4 denotes the addition of 0.2 g of PTA; MWCNTs/PVA/PTA6 denotes the addition of 0.3 g of PTA; MWCNTs/PVA/PTA8 denotes the addition of 0.4 g of PTA; and MWCNTs/PVA/PTA10 denotes the addition of 0.5 g of PTA. Five microliters of the above composite hydrogel was drop-coated onto the 0.5 cm * 0.5 cm effective area on the surface of the pre-cleaned ITO working electrode, dried at room temperature, and then cyclic freeze–thawed (one cycle includes freezing at −20 °C for 30 min and thawing at 25 °C for 30 min) to form the hydrogel/ITO sensor (Figure S1). The subsequent electrochemical test of the sensor was completed by using an electrochemical workstation and a three-electrode system.

## 3. Results and Discussion

### 3.1. Optimization of the MWCNTs/PVA/PTA Electrochemical Sensor

This study will utilize purified samples for PCN detection. By matching the retention time in HPLC and the ion peak in MS of the purified PCN sample with those of the PCN chemical reference substance (CRS PCN), we confirmed that the purified sample is indeed PCN and suitable for subsequent sensing experiments (Figure S2). The sensors prepared with MWCNT dispersion exhibited non-uniform block shedding of the material during testing; similarly, the MWCNTs/PVA sensor demonstrated shedding of the composite membrane after absorbing water and swelling. As depicted in Figure S3, neither of these sensors could achieve precise detection of PCN. Hence, there is an urgent necessity to develop stable hydrogel sensors based on MWCNTs.

The prepared MWCNTs/PVA/PTA hydrogels were found to exhibit two significant issues. Firstly, there is a clear correlation between the state of the hydrogel film and its standing time. OM observations revealed that the films prepared by drop coating after one or two days of standing exhibited noticeable agglomeration. However, this reunification situation significantly improved when the standing time exceeded three days (Figure S4). Secondly, the stability of MWCNTs/PVA/PTA hydrogel films was closely associated with the content of PTA. Based on these phenomena, this study initially investigated the impact of hydrogel resting duration, the introduction of PTA stabilizer, and the number of freeze–thaw cycles on the sensors and their associated mechanisms. As shown in Figure S5A, hydrogels that had been rested for at least 4 days exhibited stabilized response currents without any surface agglomeration. The disappearance of agglomeration can be attributed to the two-stage process of PVA dissolution—swelling and dissolution. The dissolution process is relatively slow. Even when PVA particles are no longer visible to the naked eye and then stirring ceases, the dissolution process remains incomplete. At this stage, the internal PVA chains are not fully stretched, and they still appear to be coiled or intertwined. With increasing resting time, the PVA chains have more opportunity to adjust and rearrange, thereby reducing agglomeration. Additionally, ultrasonic treatment of MWCNTs/PVA or MWCNTs/PVA/PTA solutions prior to electrode modification further promotes the unwinding of PVA chains. The quality of the modified hydrogel film is influenced by different qualities of PTA mixed with PVA, which also affects the current response. PTA2 shows slight swelling and shedding over prolonged periods; however, PTA4 and PTA6 do not have such issues. On the other hand, both PTA8 and PTA10 deteriorate in terms of hydrogel film quality. The increased PTA content adversely affects the mechanical properties of PVA films. The toughness of polymer film diminishes when PTA content in the composite system is excessively high, leading to an increased propensity for cracking [24]. By synthesizing response currents as presented in Figure S5B, we chose PTA4-modified electrodes. Additionally, it was observed that multiple freeze–thaw cycles increase density, hindering the electron transport process [25]; therefore we investigated how different numbers of freeze–thaw cycles affect sensor performance, revealing that two cycles yielded optimal current response (Figure S5C). In summary, sensors with MWCNTs/PVA/PTA4 hydrogels drop-coated onto ITO and subjected to two freeze–thaw cycles were chosen for subsequent testing and mechanistic analysis after a resting period of four or more days following preparation.

### 3.2. Mechanism of Hydrogel Membrane Adhesion Enhancement of Electrochemical Sensor

**Figure 1:**
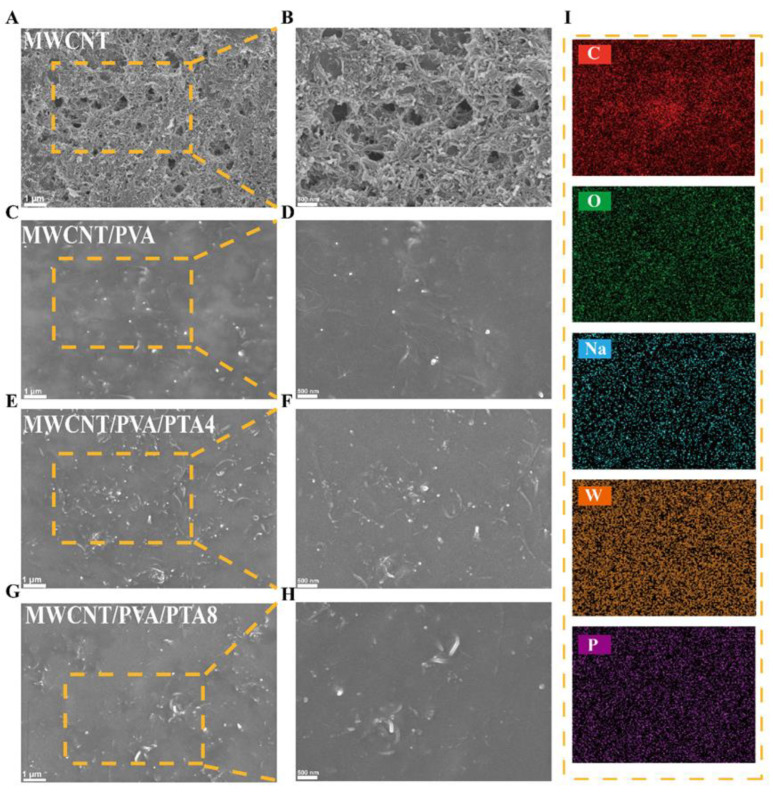
SEM and element mapping images of modified electrochemical sensor. (**A**,**B**) MWCNTs; (**C**,**D**) MWCNTs/PVA; (**E**,**F**) MWCNTs/PVA/PTA4; (**G**,**H**) MWCNTs/PVA/PTA8; (**I**) the element mapping images of the MWCNTs/PVA/PTA4.

The SEM observations of the sensor surface morphology of different hydrogel systems are presented in Figure 1. As depicted in Figure 1C,D, compared to MWCNTs with a surface consisting of numerous clustered and intertwined strip MWCNTs (Figure 1A,B), the surface of MWCNTs/PVA appears smooth, indicating clear film-forming effects. This suggests that PVA physically cross-links to form a hydrogel during freeze–thawing, while the decrease in visible MWCNTs on the surface at the same scale is likely due to their encapsulation within the hydrogel matrix. Conversely, upon the addition of varying ratios of PTA within the system, an increasing number of MWCNTs can be observed on the surface. The film-forming quality of the MWCNTs/PVA/PTA4 surface decreased, resulting in increased exposure of MWCNTs and a higher number of accessible active sites (Figure 1E,F). From Figure 1I and Figure S6, it can be observed that MWCNTs, PTA, and SDS dispersant within the hydrogel exhibit more uniform distribution on the electrode surface. Interestingly, further increasing PTA content within the system (Figure 1G,H) does not result in a continued increase in surfaced MWCNTs as seen with MWCNTs/PVA/PTA4; instead, regional agglomeration occurs. It is hypothesized that after an increase in PTA content, PTA itself becomes enriched and causes agglomeration of MWCNTs specifically in this region while deteriorating homogeneity of the surface material.

**Figure 2:**
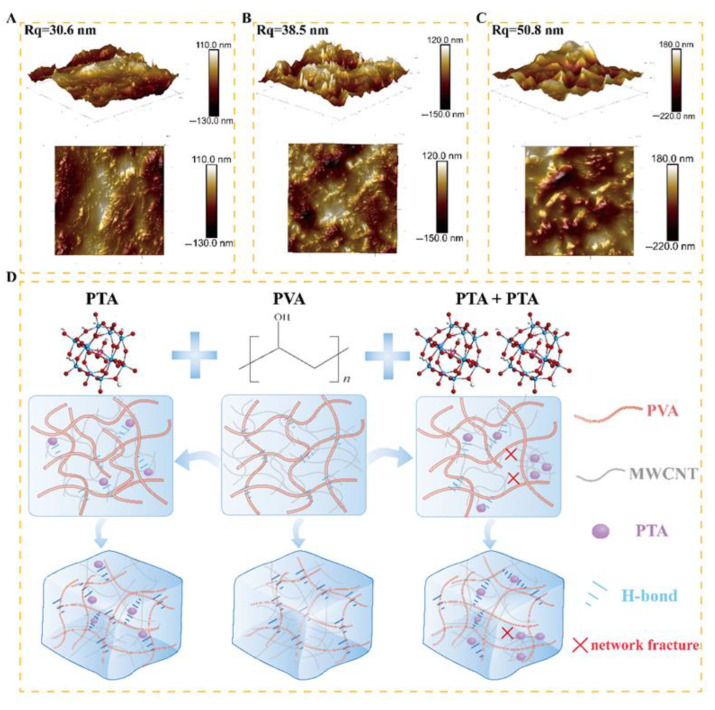
3D and 2D AFM image of modified electrochemical sensor. (**A**) MWCNTs/PVA; (**B**) MWCNTs/PVA/PTA4; (**C**) MWCNTs/PVA/PTA8; (**D**) possible mechanism diagram of the sensor.

The AFM test results also support the above analysis. Figure 2A illustrates the surface morphology of MWCNTs/PVA with an R_q_ value of 30.6 nm, revealing the embedded MWCNTs within the hydrogel in its 2D morphology. Following the addition of PTA, there was a varying degree of increase in roughness on the material surface. As depicted in Figure 2B, the R_q_ value of MWCNTs/PVA/PTA4 increased to 38.5 nm, and a sharp peak different from that of MWCNTs/PVA was obviously observed on the 3D; additionally, there was an increase in granularity on its surface as evident from its 2D morphology. These changes can be attributed to a decrease in the crystallinity of PVA after incorporating PTA and subsequent re-exposure of MWCNTs. Figure 2C displays the surface morphology of MWCNTs/PVA/PTA8, where doubling the content of PTA led to a rise in R_q_ to 50.8 nm; moreover, sharp small peaks present in MWCNTs/PVA/PTA4 were replaced by smooth large peaks observed through its 3D morphology, and visibility of exposed MWCNTs was limited in 2D morphology, aligning with the SEM speculation that PTA enrichment results in MWCNT clustering within this region.

The adhesion enhancement principle and conductivity mechanism of the fabricated sensors were analyzed in combination with SEM and AFM results. As depicted in Figure 2D, upon the introduction of PTA, its terminal oxygen ion O_b_ and bridging oxygen ligand O_c_ establish hydrogen bonds with the hydroxyl group on the side chain of PVA, thereby reducing the crystallinity of PVA and alleviating internal stress during cross-linking. Consequently, detachment issues between MWCNTs/PVA films during testing are resolved. The SDS-mediated dispersion process of MWCNTs leads to the connection between the side walls of MWCNTs by alkyl chains with negatively charged groups. Moreover, due to electrostatic effects, the negatively charged PTA molecule anchors itself to the side-walls of MWCNTs away from their negatively charged head-groups [26]. Simultaneously forming hydrogen bonds with PVA, PTA bridges both the MWCNT network and the PVA network to form a conductive network (MWCNTs/PVA/PTA), facilitating rapid proton transport via hydrogen bonding as proposed by the Grotthuss effect [27]. Furthermore, the addition of PTA enhances sensing performance by increasing accessible active sites on the sensor surface through the re-exposure of MWCNTs. With agglomeration of PTA in MWCNTs/PVA/PTA8, fractures in the conductive network structure occur, which inhibits the proton transport process dictated by the Grotthuss effect, leading to a decrease in electrical conductivity [28]. Meanwhile, following PTA agglomeration, the terminal oxygen ions of PTA are hindered from forming hydrogen bonds with the hydroxyl groups of PVA, thereby weakening PTA’s ability to inhibit PVA crystallization. Compared to MWCNTs/PVA/PTA4, the crystallinity of MWCNTs/PVA/PTA8 and MWCNTs/PVA/PTA10 increased, resulting in more brittle films and poorer overall quality during testing.

**Figure 3:**
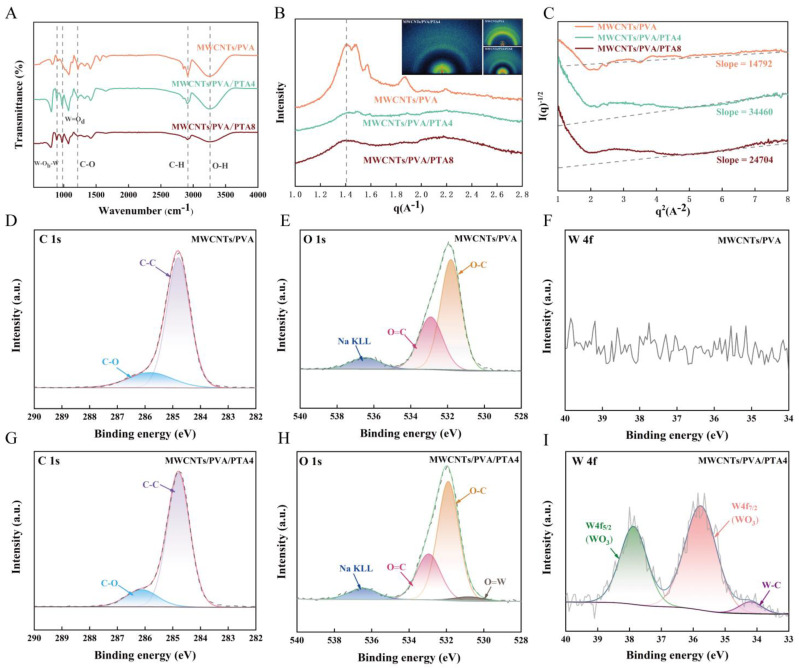
(**A**) FT-IR of modified electrochemical sensor; (**B**) WAXS of modified electrochemical sensor; (**C**) I(q)^−1/2^-q^2^ curves of modified electrochemical sensor; (**D**) C 1s XPS spectrum of MWCNTs/PVA; (**E**) O 1s XPS spectrum of MWCNTs/PVA; (**F**) W 4f XPS spectrum of MWCNTs/PVA; (**G**) C 1s XPS spectrum of MWCNTs/PVA/PTA4; (**H**) O 1s XPS spectrum of MWCNTs/PVA/PTA4; (**I**) W 4f XPS spectrum of MWCNTs/PVA/PTA4.

The XRD results of hydrogel-modified electrodes from different systems are presented in Figure S7, where the intensity of PVA crystallization peaks gradually decreases and eventually disappears with increasing PTA content [29]. Additionally, it is noteworthy that the diffraction peaks corresponding to each crystal plane of the ITO substrate become smaller upon the addition of PTA [30]. This phenomenon can be attributed to a larger uncovered area on MWCNTs/PVA-modified electrodes. The inclusion of PTA reduces the surface tension of the hydrogel, leading to better coverage during the drop-coating process. In comparison to MWCNTs/PVA, distinct peaks related to PTA can be observed in XRD results for MWCNTs/PVA/PTA4. However, these peaks weaken in MWCNTs/PVA/PTA8 due to increased concentration and potential disruption caused by enrichment interfering with periodic arrangement across the entire surface. FT-IR was employed for further understanding of the internal state of the three hydrogel systems. As depicted in Figure 3A, the introduction of PTA resulted in the appearance of two stretching vibration peaks at 981 cm^−1^ (W=O_d_) and 896 cm^−1^ (W-O_b_-W) on the infrared spectrum, indicating successful intervention of PTA in the hydrogel system [31,32,33]. The asymmetric stretching vibration peaks belonging to C-O at 1215 cm^−1^ gradually decreased with the addition of PTA, which proved that PTA hindered the crystallization of PVA and consequently reduced C-O within the conductive hydrogel network.

The phase separation morphology of the blended films was analyzed using the WAXS technique. As shown in Figure 3B, the addition of PTA resulted in broadening of the characteristic peaks. In MWCNTs/PVA/PTA4, the characteristic scattering peaks shifted towards larger q values, indicating a reduction in phase separation size between PTA and PVA after blending and formation of a homogeneous blended phase. However, for the MWCNTs/PVA/PTA8 system, the characteristic peaks shifted towards smaller q regions with an increase in phase separation size between PTA and PVA, leading to poor fusion between them as observed by SEM analysis. Both inset and Figure S8 suggest that MWCNTs/PVA exhibit low crystallinity, which is enhanced by doping with an appropriate amount of PTA. Further data analysis was performed using Debye–Beuche model fitting as shown by the Debye–Beuche equation [34,35]:

(1) Iq−12=KaQq2+Ka3Q,

where *K* is a constant, *a* represents coherence length, and *Q* denotes scattering invariant. As presented in Figure 3C, the slope of the straight line for MWCNTs/PVA/PTA4 is greater than that of MWCNTs/PVA/PTA8, indicating a smaller coherence length for the former system compared to the latter. This observation suggests that MWCNTs/PVA/PTA4 exhibits a superior blending effect.

The surface chemical bonding states of the sensors were analyzed using XPS. According to the XPS full spectra (Figure S9), it is evident that both MWCNTs/PVA and MWCNTs/PVA/PTA4 exhibit characteristic peaks corresponding to the elements C, O, Na, and S, while MWCNTs/PVA/PTA4 contains additional characteristic peaks of the element W. Furthermore, Figure 3D–I presents the XPS spectra of C 1s, O 1s, and W 4f for MWCNTs/PVA and MWCNTs/PVA/PTA4. From the C 1s spectra shown in Figure 3D,G, similar results are observed; typical bonds such as C-C from PVA and carboxyl group C-O from functionalized MWCNTs are evident. The O 1s spectra in Figure 3E,H reveal typical bonds, including O=C from carboxyl groups and O-C from PVA. Additionally, the O 1s spectra of MWCNTs/PVA/PTA4 showed O=W typical bonds originating from PTA. In contrast, when examining the W 4f spectra shown in Figure 3F,I, no relevant bonding of W is observed for MWCNTs/PVA alone. However, the presence of binding energy peaks associated with self-orbital splitting of WO_3_ in MWCNTS/PVA/PTA, along with the existence of typical W-C bonds, suggests that the process of PTA anchoring has partially loaded onto carbon nanotubes [36,37]. This finding also provides an explanation for the AFM results, where the self-enrichment process of PTA leads to the formation of clusters of MWCNTs.

### 3.3. Electrochemical Behavior of Electrochemical Sensor

**Figure 4:**
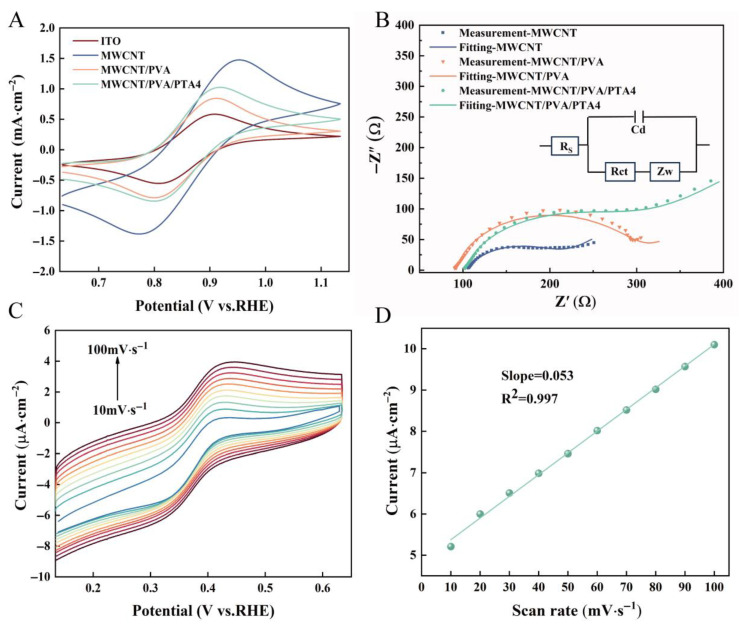
(**A**) CV characterization of modified electrochemical sensor in 5 mM [Fe(CN)_6_]^3−/4−^ and 0.1 M KCl; (**B**) EIS characterization of the modified electrochemical sensor in 5 mM [Fe(CN)_6_]^3−/4−^ and 0.1 M KCl, Randle equivalent circuit (inset 1); (**C**) CV of 50 μM pyocyanin in 1× PBS solution (pH 7.3) measured on an MWCNT/PVA/PTA4 at the different scan rates; (**D**) redox peak current of pyocyanin as a function of the scan rate.

The electrochemical behavior of the different sensors was characterized using cyclic voltammetry (CV). The CV responses of bare ITO, MWCNTs, MWCNTs/PVA, and MWCNTs/PVA/PTA4 were measured in [Fe(CN)_6_]^3-/4-^ solution. As shown in Figure 4A, the bare ITO exhibited a relatively weak redox peak compared to electrodes modified with composite materials. In contrast to the bare electrode with an oxidation peak current of 0.57 mA, the oxidation peak currents for MWCNTs/PVA and MWCNTs/PVA/PTA increased to 0.82 mA and 1.16 mA, respectively. Interestingly, the oxidation peak current of MWCNTs is 1.47 mA, which is remarkably higher than the other three curves. The peak current of the electrodes modified with materials is higher than that of bare ITO, which proves that the working electrode modified with functional materials has better conductivity. However, as the peak current of the MWCNT sensor increases, the peak-to-peak separation (Δ*E_P_*) becomes significantly greater compared to that of other electrodes, suggesting a reduction in the number of electrons transferred during the reaction as observed from Equation (2):

(2) $$\Delta E_{P}=\frac{2.3RT}{nF}\approx \frac{0.059}{n}\;\;\;,$$

where *R* is the gas constant, *T* is the temperature, *F* is the Faraday constant, and *n* is the number of electrons transferred during the redox process [38]. The electrocatalytic capacity of bare ITO, MWCNTs, MWCNT/PVA, and MWCNT/PVA/PTA4 toward PCN was measured by square wave voltammetry (SWV). As shown in Figure S10, the results show that all four electrodes have the ability to electrocatalyze PCN. Under the same conditions, I_MWCNTs_ > I_MWCNTs/PVA/PTA4_ > I_MWCNTs/PVA_ > I_ITO,_ which was consistent with the CV results.

The electron transport characteristics of the sensors were investigated using EIS. Figure 4B shows the EIS responses of different sensors, with the Randel equivalent circuit illustrated in the inset. R_ct_ represents the charge transfer resistance, Warburg impedance (Z_W_) reflects ion diffusion behavior in the electrodes, R_S_ denotes intrinsic resistance of the test solution, and C_d_ reflects the double layer capacitance. According to the Nyquist diagram, MWCNTs exhibited a lower R_ct_ value (108.6 Ω) compared to MWCNTs/PVA/PTA4 (192.2 Ω) and MWCNTs/PVA (204.8 Ω). The formation of a hydrogel by PVA acts as an additional barrier preventing [Fe(CN)_6_]^3-/4-^ from reaching the sensor surface, resulting in poorer electron transfer capability and nearly doubled R_ct_ compared to MWCNTs. On this basis, PTA was added to MWCNTs/PVA, which reduced the crystallinity of PVA, improved electrical conductivity, and subsequently reduced R_ct_ values. The electrochemically active surface area (ECSA) of different electrodes was further evaluated by the double-layer capacitance method. As shown in Figure S11A–E, compared with ITO, MWCNTs/PVA and MWCNTs/PVA/PTA4, MWCNTs/ITO exhibits the largest ECSA. The addition of PVA-encapsulated MWCNTs in the hydrogel matrix results in a significant reduction in active sites. The addition of PTA caused re-exposure of MWCNTs, and the active sites of MWCNTs/PVA/PTA4 increased compared with MWCNTs/PVA, which was consistent with SEM and AFM results.

The variable scan speed CV test was performed by maintaining a fixed CV scanning range while altering the scanning rate in a PBS solution containing 50 µM PCN. As depicted in Figure 4C,D, both the anodic and cathodic peak currents (*I_p_*) of the redox reaction exhibited a gradual increase with increasing scanning rate. Moreover, the magnitude of these current values displayed a linear relationship with the scanning rate (R^2^ = 0.997). This observation suggests that the detection of PCN on MWCNTs/PVA/PTA4 sensors follows a typical adsorption-controlled process as described by Equation (3):

(3) $$I_{p}=\frac{n^{2}F^{2}}{4RT}\upsilon A\Gamma^{*}\;\;\;,$$

where *υ* is the CV scan rate, *A* is the electrode area, and Γ* is the surface coverage of the adsorbed substance [39]. The sensor was subjected to subsequent electrochemical testing after being incubated for the same duration in solutions with varying concentrations of the target substances, following the characterization of the adsorption reaction.

### 3.4. Sensing Performance of the MWCNTs/PVA/PTA4 Sensor

**Figure 5:**
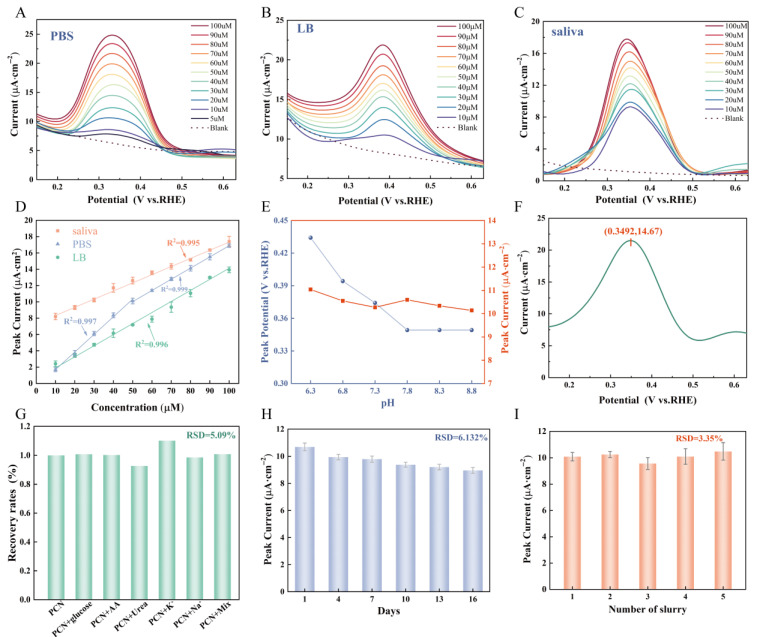
(**A**) SWV current response of PCN in the range of 5–100 μM in PBS solution; (**B**) SWV current response of PCN in the range of 10–100 μM in LB media; (**C**) SWV current response of PCN in the range of 10–100 μM in artificial saliva media (**D**) calibration curves of the three media. The error bar represents the standard deviation across triplicate measurements; (**E**) SWV current response and peak potential of PCN in different pH PBS solution; (**F**) SWV current response of PA culture medium supernatant(include PCN); (**G**) selective SWV response of MWCNTs/PVA/PTA4 towards 50 µM PCN in PBS solution with the presence of identical concentration of interfering; (**H**) Stability of MWCNTs/PVA/PTA4 for determination of 50 µM PCN; (**I**) reproducibility of MWCNTs/PVA/PTA4 prepared from different batches of hydrogel.

The sensing performance of the sensor in measuring PCN concentration under different electrolyte environments was evaluated by using the highly sensitive and mature SWV. As shown in Figure 5A, the oxidation peak appeared at 0.34 V when tested in PBS solution, and its oxidation peak current exhibited a good linear correlation with PCN concentration within the ranges of 5 μM~50 μM and 50 μM~100 μM (Figure S12 and Figure 5D), with correlation coefficients R^2^ of 0.997 and 0.999, respectively. The LOD of this sensor in PBS solution is 1.67 μM, which covers the range of PCN concentration of 5–100 μM in clinical infections [40]. Notably, the standard curve displayed a lower slope at higher concentrations compared to that observed at lower concentrations, suggesting a reduced sensitivity towards elevated analyte levels. This phenomenon may be attributed to either the formation of a thicker analyte layer on the electrode surface hindering analyte diffusion into the electroactive center or continuous detection starting from low concentrations resulting in partial occupation of active sites by analytes or their products while reducing surface capacity for adsorption of target substances [41]. Given that PCN serves as a marker secretion for PA, a similar test was conducted using LB broth, a commonly used medium for PA cultivation. As depicted in Figure 5B, the results of the test in LB broth exhibited the same pattern as PBS solution, with PCN oxidation behavior determined at 0.384 V. The oxidation peak current increased with increasing concentration. Its LOD is 2.71 µM. Considerations of the prevalent detection of PA colonization in lung infection sputum samples prompted us to conduct sensing tests utilizing artificial saliva, which closely mimics the composition of sputum (Figure 5C). Within this setting, the oxidation peak potential for PCN was observed at 0.354 V. The concentration standard curve exhibited an R^2^ value of 0.995 and an LOD of 3.63 µM. The sensor sensitivity in both LB broth and artificial saliva was relatively diminished compared to that in the PBS solution environment. This reduction could be attributed to the presence of proteins, peptides, and ions as components of these two electrolytes, which may influence the adsorption behavior of the tested substance. Furthermore, the salt concentration in LB broth is higher than that in PBS solution, resulting in a slightly greater viscosity in LB broth compared to PBS solution [42]. And the viscosity of artificial saliva is significantly higher than that of both LB broth and PBS solution. This increased electrolyte viscosity hinders the movement of PCN within the electrolyte, thereby reducing the adsorption of PCN onto the sensor surface and consequently decreasing sensitivity.

Variable peak potentials for PCN oxidation were detected across three distinct electrolyte conditions, suggesting a potential influence of environmental pH. Given that the pH range for chronic wounds typically falls between 7 and 9 [43], PBS solutions with varying pH values were formulated to examine the redox behavior of PCN (with a fixed concentration of 50 μM), as shown in Figure S13 and Figure 5E. The study revealed that the oxidation peak potential of PCN exhibited a pH-dependent shift. In an acidic environment, the potential was elevated, yet the discrepancy in peak currents was minimal (RSD = 3.05%), thus indicating that the sensor developed herein can exclude the influence of the test environment pH. Moreover, the concentration of PCN was determined in the supernatant from LB medium (pH = 7.9) cultured with PA bacteria. As shown in Figure 5F, the corresponding oxidation peak was located at 0.349 V, and the concentration was estimated to be 107 μM based on the established standard curve, which slightly differs from the measurement of 113 μM obtained via alternative methods (weighing after purification). Collectively, these findings underscore the utility of our sensor for practical applications.

The sensor should have excellent anti-interference capability in practical applications. Consequently, the selectivity of the MWCNTs/PVA/PTA4 sensor towards PCN was investigated in the single and mixed presence of various organic and inorganic substances such as Na^+^, K^+^, AA, urea, and glucose. As shown in Figure 5G and Figure S14, the introduction of equivalent concentrations of interfering agents into the PCN-containing PBS solution and LB medium did not result in any current deviations exceeding ±10%. To assess the stability of the sensors, those prepared on a given day were evaluated after being stored in air for different days. The current retained 83.81% of its initial value by the sixteenth day, underscoring the sensors’ long-term stability (Figure 5H). The reproducibility was appraised by utilizing disparate batches of hydrogels for sensor fabrication. As illustrated in Figure 5I, the current responses of the sensors derived from five distinct batches of hydrogels showed variations of less than ±5% under identical conditions. The MWCNTs, PVA, and PTA within the hydrogel system were all highly stable and nonvolatile in the air environment [44], resulting in good stability and reproducibility of the sensors.

## 4. Discussion

In this study, MWCNTs/PVA/PTA hydrogels were prepared and applied to the biosensing field for the first time. The mechanism by which PTA modulates the adhesive properties and sensing sensitivity of the hydrogel is elucidated. The developed sensor was employed for the detection of PCN in diverse electrolyte environments, including PBS solution, LB, and artificial saliva, exhibiting a linear detection range of 5–100 μM and an LOD of 1.67 μM in PBS solution. More importantly, it was found that PTA inhibited the low adhesion of PVA caused by internal stress, promoted the proton transport efficiency in the conductive network, and expanded the application range of MWCNTs/PVA/PTA sensors in various solution environments. Hence, the MWCNTs/PVA/PTA4 sensor fabricated in this research demonstrates considerable potential for universal PCN detection. The proposed mechanism of PVA composite PTA opens up a new way for further in-depth study of composite hydrogel sensing films.

## Data Availability

All data generated or analyzed during this study are included in this article or supplementary material.

## Supplementary Materials

The following supporting information can be downloaded at: https://www.mdpi.com/article/10.3390/s25020557/s1. The file includes methods and additional data. Figure S1: Schematic diagram of MWCNTs/PVA/PTA4 sensor structure and test process; Figure S2: HPLC and MS comparison of the CRS and purified PCN; Figure S3: Calibration curves of different material system modified sensor; Figure S4: OM image of modified sensor at different day; Figure S5: Optimization of the modified sensor. Figure S6: SEM and EDX image of MWCNTs/PVA/PTA4; Figure S7: XRD pattern of different material system modified sensor; Figure S8: WAXS 2D pattern of different material system modified sensor; Figure S9: XPS survey spectra of different material system modified sensor; Figure S10: SWV current response of different material system modified sensor; Figure S11: ECSA results of different material system modified sensor; Figure S12: Calibration curves of PBS solution; Figure S13: SWV current response of PCN in different pH PBS environment; Figure S14: SWV current response of PCN in LB medium.
