# Supplementary material for: Development of Robust MWCNT Hydrogel Electrochemical Biosensor for Pyocyanin Detection by Phosphotungstic Acid Modification
Source: Sensors (Basel). 2025 Jan 19;25(2):557. doi: 10.3390/s25020557 (PMC11769391; doi:10.3390/s25020557)
Supplement: Supplementary file 1 [file sensors-25-00557-s001.zip › sensors-3402227-supplementary.pdf]

SUPPORTING INFORMATION

Development of Robust MWCNTs Hydrogel Electrochemical Biosensor for Pyocyanin Detection by Phosphotungstic Acid Modification

Ting Xue[1], Lei Gao[2], Xianying Dai[1,*], Shenhui Ma[1], Yuyu Bu[1,*] and Yi Wan[2,*]

[1]Key Laboratory of Wide Band-Gap Semiconductor Materials and Devices, School of Microelectronics, Xidian University, Xi'an 710071, China

[2]Microbiology Institute of Shaanxi, No.76 Xiying Road, Xi'an 710043, China

SUPPLEMENTARY METHODS

Materials

Carboxylated multi-walled carbon nanotubes (>50 nm) were purchased from Jiangsu XianFeng Nano Technology Co.,Ltd.,China.; sodium decyl sulfate(SDS),poly(vinyl alchol)(PVA, MW 146000-186000,with 99% degree of hydrolysis), phosphotungstic acid hydrate(H3O40PW12·xH2O), pyocyanin CRS, were obtained from Aladdin Chemical Reagent Co., Ltd., China. Pyocyanin sample purified from P. aeruginosa. All solutions were prepared using Milli-Q grade water (Millipore water purification system Z18 MΩ, Milli-Q, Millipore, Billerica, MA).

Instruments

The microscopic morphology and and elemental mapping of the sensor was observed by scanning electron microscope(SEM, GeminiSEM 360, Zeiss, Germany) and optical

microscope(OM,Lab A1,Zeiss, Germany); Atomic force microscope (AFM, Dimension Icon, Bruker, America) was used to characterize the electrode surface micrographics with a scan frequency of 1.58 Hz; The chemical composition and crystal structure of the electrode were characterized by X-ray diffraction analysis (XRD, D8 Advance, Bruker, America) ,X-ray photoelectron spectroscopy (XPS, ESCALAB Xi+, Thermo Scientific, America) and Fourier Transform infrared spectroscopy(FT-IR, IRTracer 100, Tsushima Co.,Japan); wide angle X-ray scattering (WAXS, Xeuss 2.0, Xenocs,French) was used to characterize degree of integration of the two phases.

Electrochemical performance measurements were performed at a ChenHua Electrochemical Workstation (CHI660E, Chenhua Instrument Co., Ltd., China) in a traditional three-electrode cell containing 1x phosphate buffer solution (PBS, pH 7.3) with Ag/AgCl as reference electrode and a piece of platinum as counter electrode, respectively. electrochemical impedance spectroscopy (EIS) was completed in a solution of 0.1 M KCl containing 5 mM $[Fe(CN)_6]^{3-/4-}$ at a Zahner Zennium pro Electrochemical Workstation (Zahner, Germany) in the same three-electrode cell.

The detection limit (LOD) of sensor can be calculated by the formula:

$$LOD = \frac{3S_b}{K}$$

where S_b is standard deviation of blank sample, k is slope of the calibration curve.

SUPPLEMENTARY FIGURES

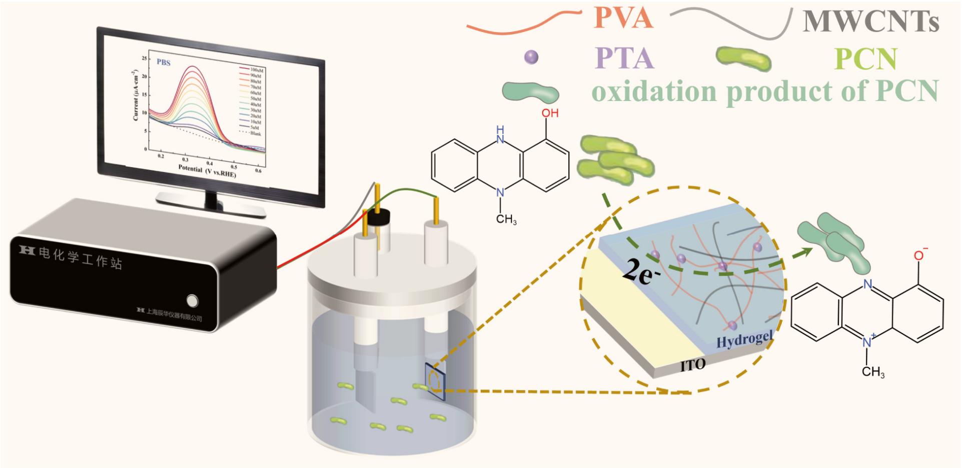

Figure S1. Schematic diagram of MWCNTs/PVA/PTA4 sensor structure and test process

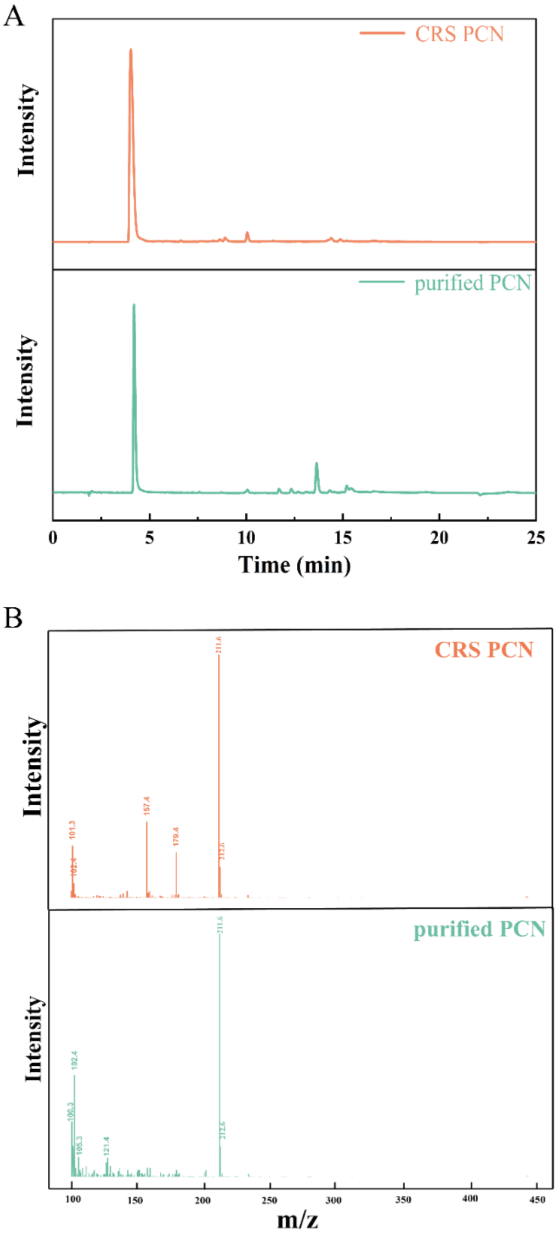

Figure S2. (A) HPLC comparison of the CRS PCN and purified PCN; (B) MS comparison of the CRS PCN and purified PCN.

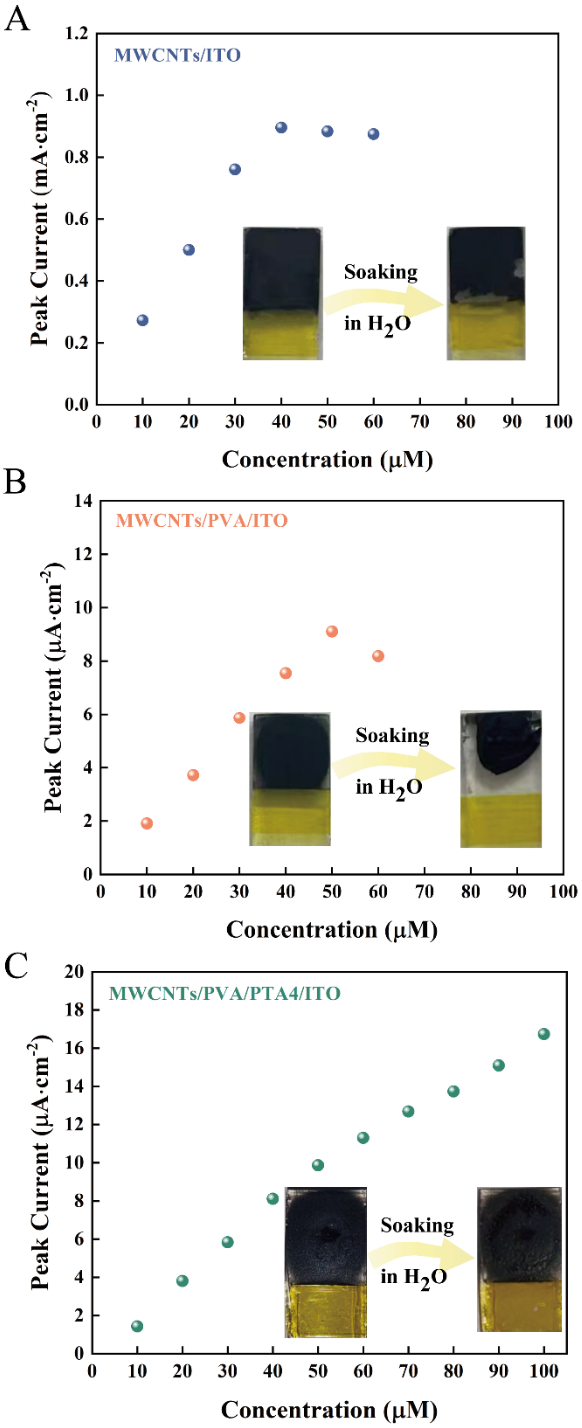

Figure S3. Calibration curves of the three different material system modified sensor (A) MWCNTs; (B) MWCNTs/PVA; (C)MWCNTs/PVA/PTA4.

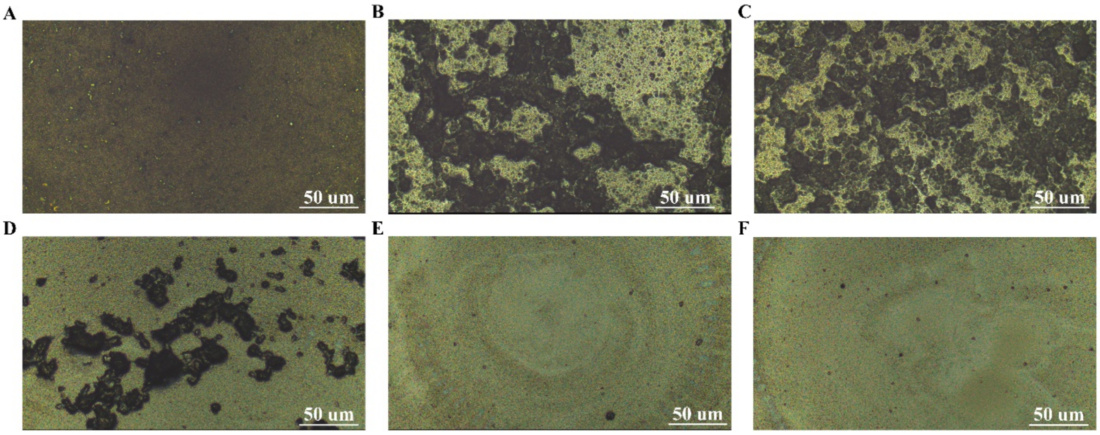

Figure S4. OM image of modified sensor at different day. (A)MWCNTs at day1; (B)MWCNTs/PVA at day1; (C) MWCNTs/PVA/PTA4 at day1; (D) MWCNTs/PVA/PTA4 at day2; (E) MWCNTs/PVA/PTA4 at day3; (F)MWCNTs/PVA/PTA4 at day4.

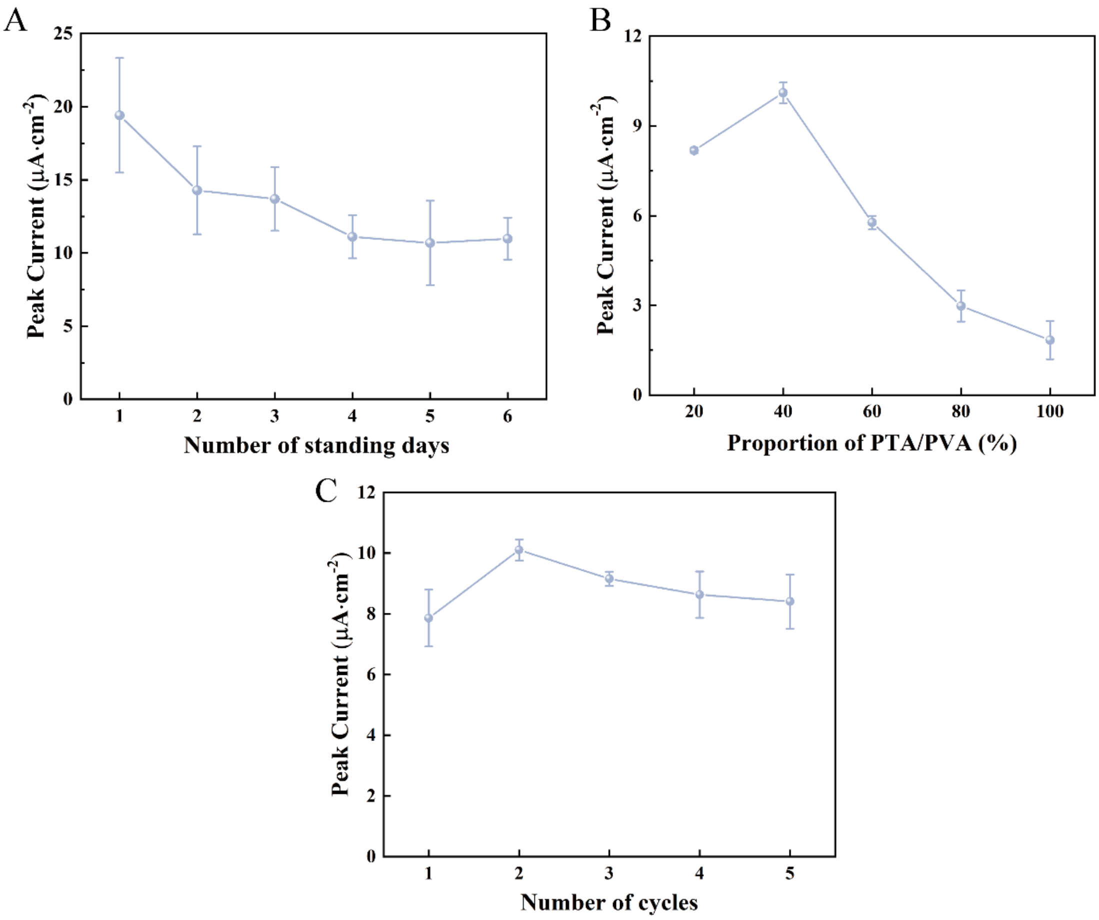

Figure S5. Optimization of the modified sensor. (A) current response of hydrogel-modified sensors with different standing days (B) current response of modified sensor at proportion of PTA/PVA; (C) current response of modified sensor at number of cycles.

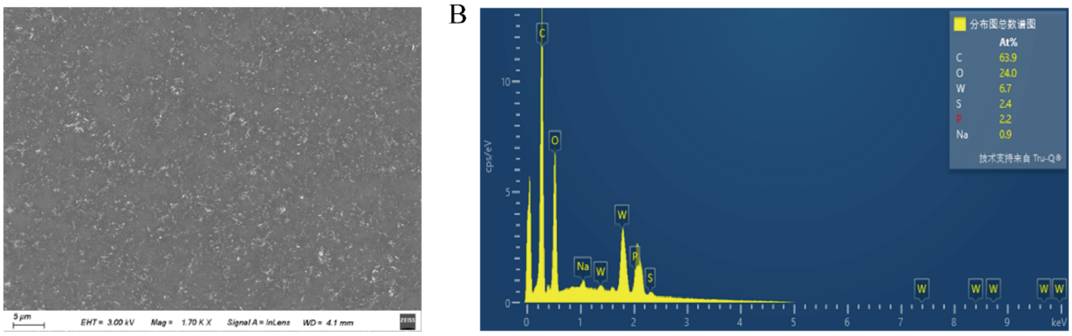

Figure S6. (A) SEM image of MWCNTs/PVA/PTA4 at the scale of 5 μm (B) EDX energy spectrum of MWCNTs/PVA/PTA4.

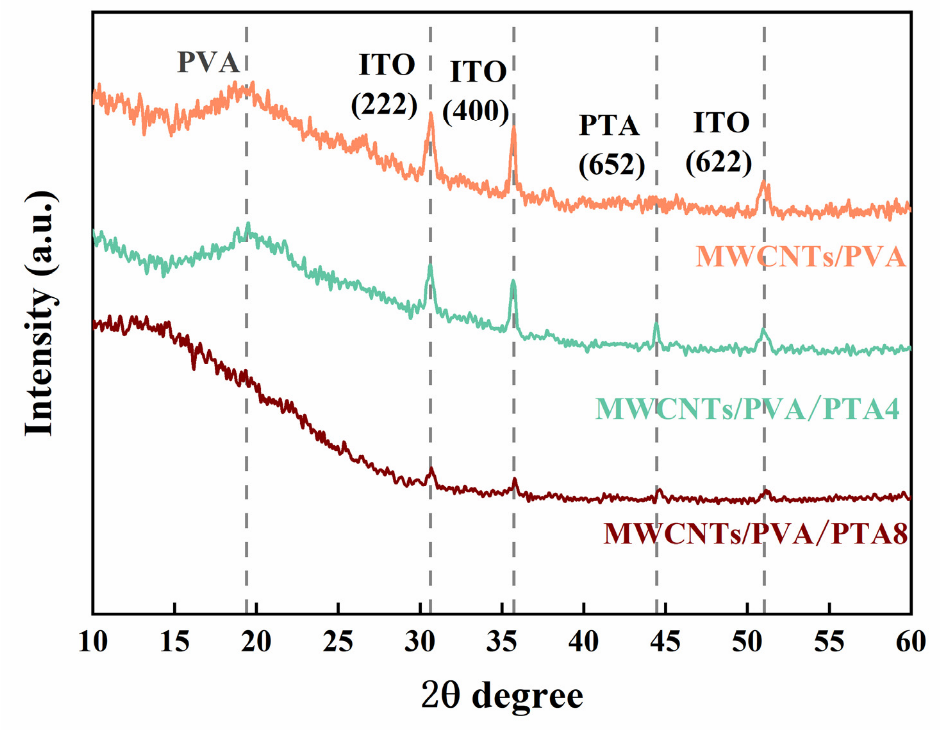

Figure S7. XRD pattern of MWCNT/PVA, MWCNT/PVA/PTA4 and MWCNT/PVA/PTA8.

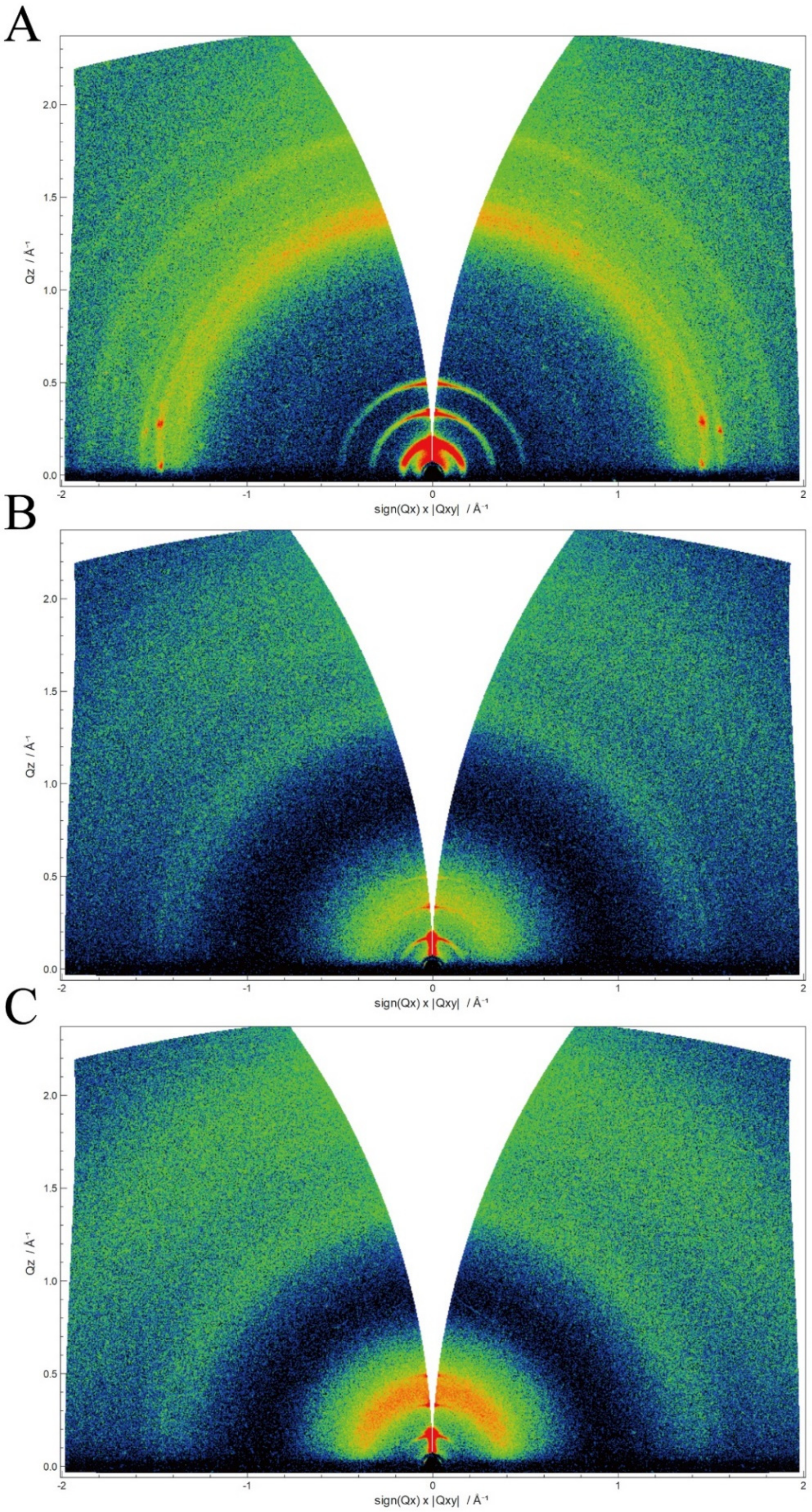

Figure S8. WAXS 2D pattern of the three different material system modified sensor (A) MWCNTs/PVA; (B)MWCNTs/PVA/PTA4; (C)MWCNTs/PVA/PTA8.

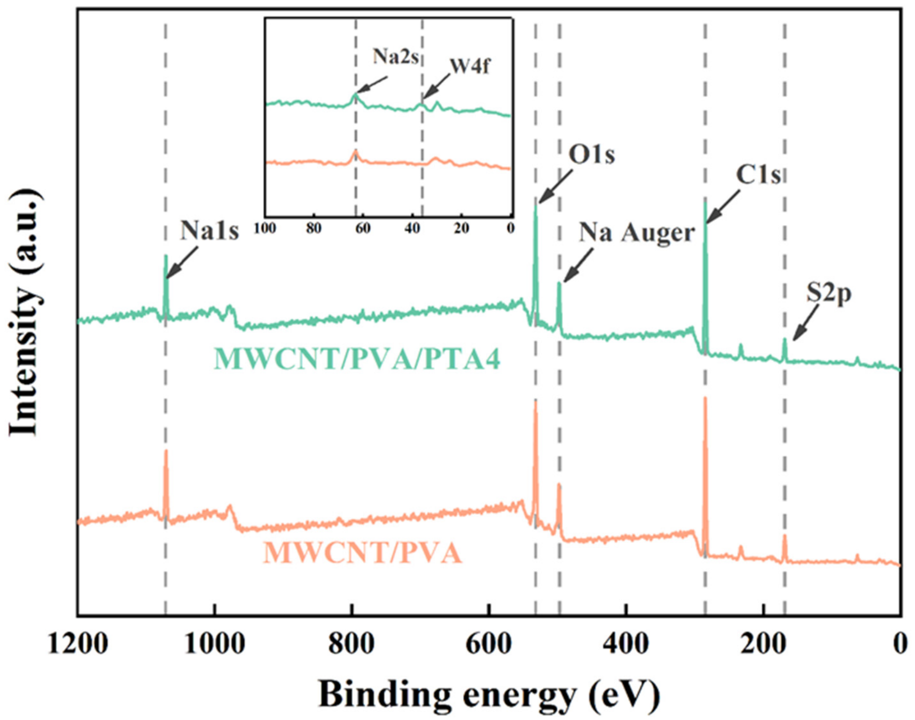

Figure S9. XPS survey spectra of MWCNT/PVA and MWCNT/PVA/PTA4.

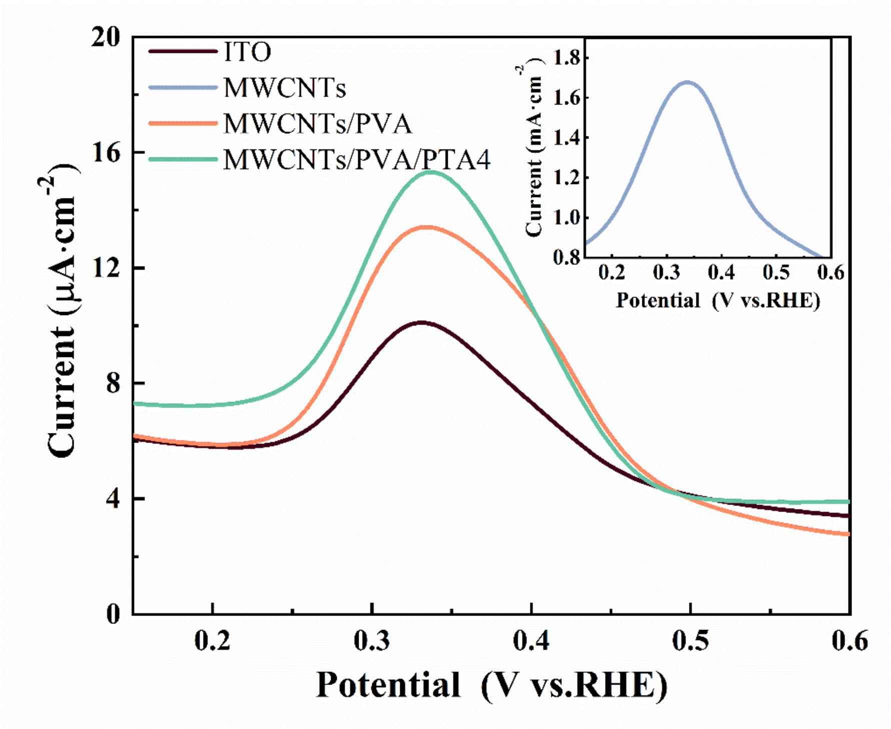

Figure S10. SWV current response of modified electrochemical sensor in PBS solution containing 50 μM PCN.

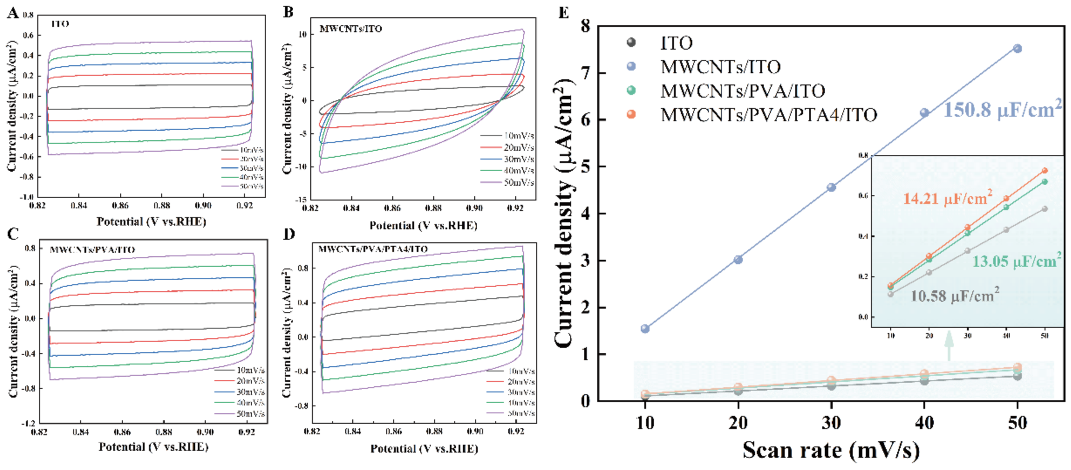

Figure S11. Cyclic voltammetry curves of modified electrodes at different scan rates (A) ITO; (B) MWCNTs/ITO; (C) MWCNTs/PVA/ITO; (D) MWCNTs/PVA/PTA4/ITO; (E) Current density difference-scan rate relationship diagram of modified electrodes.

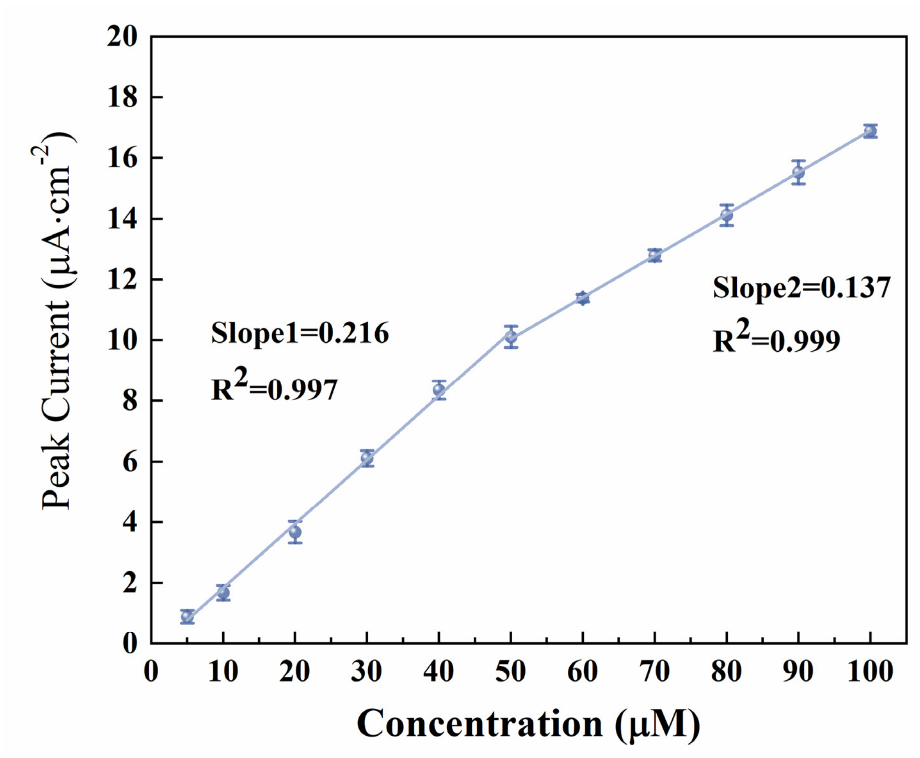

Figure S12. calibration curves of PBS solution. The error bar represents the standard deviation across triplicate measurements.

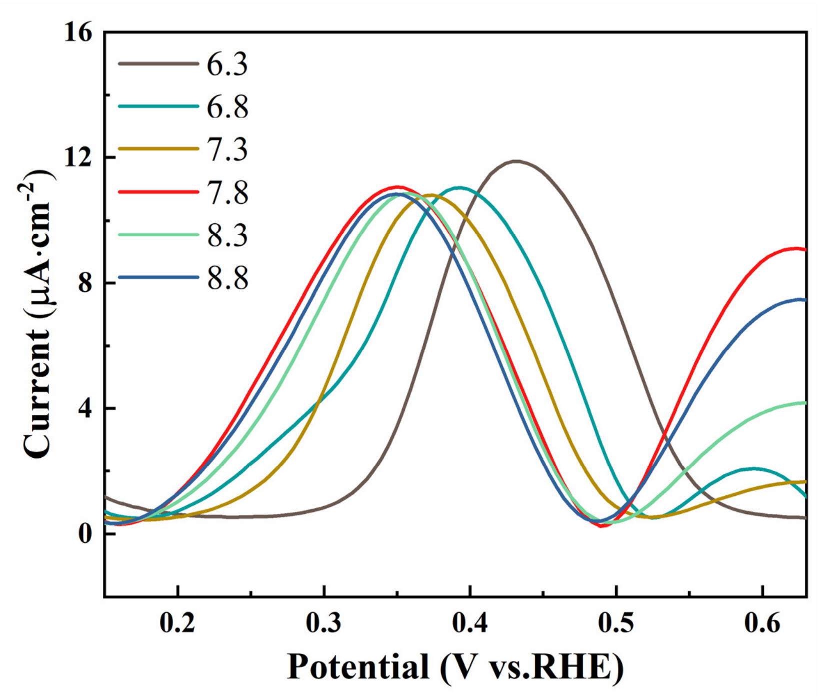

Figure S13. SWV current response of PCN in different pH PBS environment.

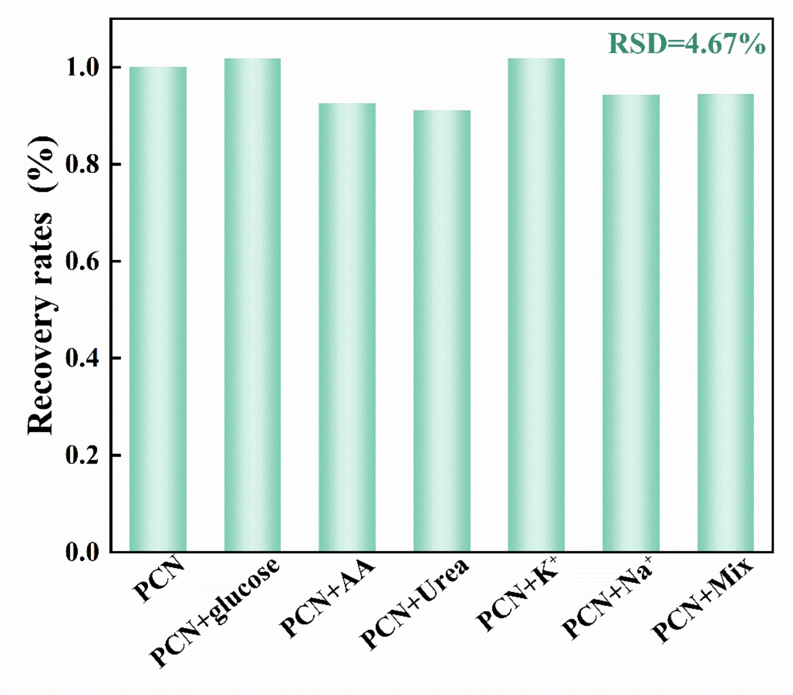

Figure S14. Selective SWV response of MWCNTs/PVA/PTA4 towards 50 μM PCN in LB medium the presence of identical concentration of interfering.
